# The cumulative contribution of direct and indirect traumas to the production of PTSD

**DOI:** 10.1371/journal.pone.0307593

**Published:** 2024-08-14

**Authors:** Dan Even, Gregory H. Cohen, Ruochen Wang, Sandro Galea

**Affiliations:** 1 Moshe Prywes Center for Medical Education, Faculty of Health Sciences, Ben Gurion University, Beer Sheva, Israel; 2 School of Public Health, Boston University, Boston, Massachusetts, United States of America; Tribhuvan University Institute of Medicine, NEPAL

## Abstract

**Objective:**

Posttraumatic Stress Disorder (PTSD) affects millions of people worldwide. While the relationship between direct exposure to traumatic events and PTSD is well-established, the influence of indirect trauma exposure on PTSD remains unclear. It is similarly unclear what role cumulative exposure to direct and indirect traumas play in the risk of PTSD.

**Methods:**

The study uses data from the Houston Trauma and Recovery Study, conducted on 2020–2021, and involved a random sampling of 1,167 individuals residing in Houston during Hurricane Harvey in 2017. Participants were asked about their experiences related to both Hurricane Harvey and the subsequent COVID-19 pandemic. Exposures were categorized as direct or indirect traumas, in line with the criteria delineated in the fifth edition of the Diagnostic and Statistical Manual of Mental Disorders (DSM-5). Cumulative exposures were also calculated.

**Results:**

Among participants, 12.6% were experiencing current PTSD. There were significant associations between both direct [OR = 3.18, 95% CI 1.85, 5.46] and indirect [OR = 1.91, 95% CI 1.05, 3.46] traumas related to Harvey, as well as direct [OR = 2.13, 95% CI 1.20, 3.77] and indirect [OR = 1.69, 95% CI 0.93, 3.09] traumas due to COVID and the risk of PTSD in fully adjusted models. Further, significant associations were found between the cumulative exposure to traumas from both Hurricane Harvey and COVID-19 and the risk of PTSD, considering both direct [OR = 2.53, 95% CI 1.36, 4.70] and indirect exposures [OR = 2.79, 95% CI 1.47, 5.28].

**Conclusions:**

Our study offers support for connections between exposure to both direct and indirect traumas stemming from large-scale disasters and PTSD. Moreover, we show that cumulative exposures to multiple large-scale events increase the risk of PTSD. This highlights the importance of the consideration of a range of exposures as risks for PTSD, particularly in a time of compounding disasters and broad population exposures to these events.

## Introduction

Posttraumatic Stress Disorder (PTSD) is a recognized psychiatric disorder affecting millions of people worldwide [1–7]. Evidence shows connections between a range of traumatic event exposures and PTSD, including exposure to inter-personal traumas (e.g., violence) and large-scale natural disasters [8, 9]. However, fundamental questions remain regarding the nature of traumatic events that can lead to PTSD.

A firmly established connection exists between direct exposure to traumatic events, such as natural disasters, life-threatening accidents, physical and sexual assaults, military combat, war exposure, life-threatening illnesses, and the traumatic loss of a loved one, and the susceptibility to PTSD [1–4, 8].

Other work has suggested that indirect traumatic events might also lead to greater risk of PTSD [10–14]. The concept of indirect trauma was initially introduced in the literature through the terms "secondary traumatization" and "vicarious traumatization," and defined as the experience of trauma indirectly affecting a family member, a close friend, a therapy client [10] or the exposure to trauma indirectly while performing professional duties, such as those carried out by emergency rescue crews [11]. In the 2013 5^th^ version of the Diagnostic and Statistical Manual of Mental Disorders (DSM-5), a change was introduced in the definition of PTSD, incorporating indirect exposure to trauma into diagnosis criterion A (Exposure Criterion), defined as the "Indirect exposure to aversive details of the trauma, usually in the course of professional duties (e.g., first responders, medics)" [15, 16]. The DSM-5 excluded other potential forms of indirect trauma, such as observing traumatic events through electronic media, television, video games, or pictures, from its definition [17–19].

It remains unclear, however, the role these indirect traumatic events play in shaping PTSD. Some researchers have raised questions about the validity of including indirect exposure in the definition of PTSD [20, 21], while others have supported the concept [3].

A second, and related question pertains to the role of additive traumatic experiences to the risk of PTSD [22, 23], and whether cumulative exposure to direct and indirect traumatic events similarly increase the risk of PTSD [24–26]. Theory and research suggest that prior encounters with traumatic events can heighten sensitivity to adverse consequences when facing subsequent potentially traumatic events [27]. Substantial evidence emerges on the cumulative exposure to childhood traumas influencing the subsequent development of PTSD [28]. Research also suggests a dose-response relationship between the number of childhood traumas and comorbid psychiatric problems, including PTSD, in certain populations [29]. Cumulative exposure models to traumatic events suggest a dose-response relationship [30–32]. To our knowledge, there has been no previous work that had specifically examined the cumulative effects of indirect traumatic exposures on PTSD.

Our study seeks to investigate the impact of exposure to both direct and indirect traumas, as well as cumulative trauma, on PTSD, within the context of Houston, Texas where residents experienced the large-scale natural disaster of Hurricane Harvey, followed by the COVID-19 pandemic.

In August 2017, Hurricane Harvey, classified as a Category 4 storm on the Saffir-Simpson hurricane scale, hit Texas and Louisiana upon reaching land, establishing itself as one of the most severe tropical cyclones in the history of the United States [33]. The hurricane led to 68 direct fatalities and 35 deaths attributed to indirect causes, causing flooding in over 300,000 structures and affecting up to 500,000 cars. Additionally, approximately 336,000 households lost power, and 40,000 flood victims were either displaced or sought refuge in shelters, resulting in an estimated total of $125 billion in damages [34].

Three years later, the COVID-19 pandemic emerged as a protracted natural disaster [35]. Alongside more than 770 million confirmed cases and nearly 7 million confirmed deaths [36], The pandemic introduced a variety of social and economic challenges that are strongly linked to PTSD within the general population [37], among those directly exposed and hospitalized [38], as well as among healthcare workers experiencing indirect exposures [39].

These two disasters presented an opportunity to study the impact of exposures to direct and indirect traumas, as well as the cumulative impact of these traumas on PTSD among individuals in Houston who faced both Hurricane Harvey and the subsequent COVID-19 pandemic.

## Materials and methods

This study examined the exposure to both direct and indirect trauma during Hurricane Harvey and the COVID-19 pandemic and explored their relationship with the probable diagnosis of PTSD.

### Study sample

The Houston Trauma and Recovery Study (HTRS) is an observational survey that employed random address-based sampling of residents from 88 neighborhoods in Houston, officially recognized by the city municipality as super-neighborhoods [40]. Out of 12,009 addresses to which recruitment letters were sent, 899 were identified as invalid. Participants were recruited from November 17^th^ 2020 to August 24^th^ 2021. Among the 11,110 eligible participants who received the letters, the study saw the participation of 1,266 individuals, yielding a comprehensive response rate of 11.4%. Out of the 1,266 participants, 1,167 were residents of Houston during Hurricane Harvey and constitute the sample for our study.

### Tools

Participants invited to take part in the study could opt to either complete a paper survey sent by mail or engage in a web-based survey. The questionnaire consisted of eight sections: Section A consisted of 25 questions on Hurricane Harvey experiences; section B consisted of 3 questions on potentially traumatic events; section C consisted of 20 questions on problems after potentially traumatic events; section D consisted of 9 questions on emotional health; Section E consisted of 5 questions on health between the Hurricane Harvey and COVID-19 pandemic; section F consisted of 30 questions on COVID-19 pandemic experiences; section G consisted of 2 questions on experiences in the last two weeks; and section H consisted of 12 demographic questions. Some of the sections included subsections. The survey was accessible in both English and Spanish.

We created intricate survey weights to consider factors such as the likelihood of inclusion in the survey, survey response probability, neighborhood size, and the number of adults per household. In the last phase of the weighting process, we adjusted these weights to match the demographic distributions of sex, race, ethnicity, age, and educational attained in the Houston Metro Area as of 2020 [41]. Additionally, to tackle the effects of missing data, we conducted multivariable multiple imputation on 15 sets of data. Subsequently, we synthesized these datasets to generate consolidated estimates, which we present [42].

### Study constructs

#### Trauma

Participants were queried about their experiences in relation to Hurricane Harvey and COVID-19. We categorized these exposures based on their direct or indirect nature adhering to the criteria outlined in DSM-5. Direct exposures encompassed experiences of directly witnessing or personally experiencing a traumatic event, whereas indirect exposures pertained to the traumatic experiences of their loved ones [12]. In constructing each of the below trauma subvariables, we summed included factors and used a median split to generate binary variables, as shown in S1 Table.

#### Harvey-related direct trauma

Direct traumas related to Hurricane Harvey included experiencing a serious injury due to the hurricane and worrying about being seriously injured or killed while being away from Houston.

#### Harvey-related indirect trauma

Indirect traumas included worries about the serious injury or death of family members or close friends, a family member or close friend suffering a serious injury, and a family member or close friend passing away.

#### COVID-related direct trauma

For COVID, the Direct Traumas included four factors: becoming seriously ill from COVID, being hospitalized due to COVID, having a fear of contracting COVID, and fearing death from COVID.

#### COVID-related indirect trauma

For COVID, indirect trauma included having family members or close friends who became seriously ill or died from COVID, as well as worrying about the possibility of family members or close friends succumbing to COVID.

#### Cumulative exposure

The cumulative exposure was defined as the sum of exposures falling within a specific category, both within Hurricane Harvey and COVID, for either direct or indirect trauma.

#### Demographics

Participants were asked to provide information on several demographic attributes. They were queried about their gender, with response options, including male, female, or ’other’. Age was collected as a continuous variable. Educational attainment was divided into two categories: high school or less, and some college or higher. Marital status options encompassed being married, divorced or separated, or never married. Ethnicity was assessed as Hispanic or non-Hispanic, while race was categorized as White, Black, Asian, American Indian or Alaska Native, and Native Hawaiian or Other Pacific Islander. These racial and ethnic categories were further grouped into White non-Hispanic, Black non-Hispanic, Other non-Hispanic, and Hispanic. Income categories for the year 2019 were delineated as follows: <$24,999; $25,000-$49,999; $50,000-$74,999; $75,000-$99,999; $100,000-$149,999; $150,000-$199,999; $200,000+. Our analysis considered both the method of survey administration (mail or web) and the range of interview dates, corresponding to two distinct recruitment periods (11/17/20-3/31/21 or 4/1/21-8/24/21). Additionally, the count of children and adults in the household was included as supplementary covariates.

#### PTSD

Criteria outlined for DSM-5 PTSD [15] is met if participants fulfilled all five DSM-5 criteria for PTSD. These criteria comprise Criterion A (traumatic event), Criterion B (reexperiencing), Criterion C (avoidance and numbing), Criterion D (negative thoughts), and Criterion E (hyperarousal).

Initially, we assessed if participants fulfilled Criterion A (traumatic event), without requiring them to tie their symptoms to a specific event, in alignment with a growing body of literature [43]. To determine eligibility for Criterion A event, participants were asked whether they had personally experienced something highly stressful or if a family member or close friend had undergone such an event. We provided participants with a range of examples, encompassing natural disasters, fires, physical or sexual assault or abuse, combat exposure, encounters with death or threats to life, severe accidents, and life-threatening illnesses.

We then used the PTSD Checklist for DSM5 to establish whether participants met all DSM5 criteria for PTSD [44]. In the subsequent inquiries regarding specific symptoms, participants were asked to consider the events they previously thought about in relation to Criterion A, as well as any other highly stressful experiences they might have encountered. Symptoms were identified as present if marked as bothering a participant at least "moderately" [45].

We then proceeded to determine if participants met Criterion B (re-experiencing) by assessing the presence of one or more re-experiencing symptoms. Subsequently, we assessed whether participants met Criterion C (avoidance and numbing) by identifying the presence of one or more of these symptoms. Criterion D (negative thoughts) was evaluated by checking for the presence of two or more symptoms outlined in Criterion D, and Criterion E (hyperarousal) was assessed by verifying two or more Criterion E symptoms. Finally, we confirmed whether participants met all five DSM-5 criteria for probable PTSD.

### Analysis

The data was analyzed in 2023. First, using descriptive statistics and chi-squared tests, we assessed whether demographics and trauma exposures are related to DSM-5 PTSD (see Table 1).

**Table 1:** Demographics (n = 1167)*.

Exposure	Category	n (%)	Current PTSD n (%)	X^2^; p
Sex	Male	408 (48.1)	36 (38.4)	5.58; <0.05
	Female	749 (50.5)	98 (55.4)	
	Other	10 (1.4)	3 (6.2)	
Education	High school or less	319 (47.9)	52 (58.5)	3.56; 0.06
	Some college or higher	848 (52.1)	85 (41.5)	
Marital Status	Divorced/Separated	338 (15.8)	53 (25.1)	5.88; <0.05
	Never Married	327 (34.0)	48 (43.9)	
	Married	502 (50.2)	36 (31.0)	
Race/Ethnicity	White non-Hispanic	425 (36.6)	28 (18.4)	6.12; <0.05
	Black non-Hispanic	301 (14.2)	51 (29.2)	
	Other non-Hispanic	101 (6.0)	12 (6.1)	
	Hispanic	340 (43.2)	46 (46.3)	
Income	≤$24,999	277 (26.4)	52 (41.3)	2.46; <0.05
	$25,000-$49,999	260 (22.1)	33 (23.7)	
	$50,000-$74,999	161 (12.7)	16 (8.1)	
	$75,000-$99,999	108 (8.7)	13 (9.4)	
	$100,000-$149,999	116 (9.6)	6 (5.3)	
	$150,000-$199,999	101 (8.3)	9 (8.4)	
	$200,000+	144 (12.3)	7 (3.8)	
Survey Administration Method	Mail	665 (48.1)	87 (58.0)	3.02; 0.08
	Web	502 (51.9)	50 (42.0)	
Date of Interview	11/17/20–3/31/21	584 (50.0)	74 (52.3)	0.16; 0.69
	4/1/21–8/24/21	583 (50.0)	63 (47.7)	
Current Depression	Yes	63 (5.8)	-	-
Current PTSD	Yes	137 (12.6)	-	-
	No	1030 (87.4)	-	-

*Frequencies are unweighted; percentages, means and statistical measures are weighted

Next, we estimated multivariable logistic regressions with interaction tests to predict the adjusted associations between each category of trauma and PTSD, as shown in Tables 2 and 3. These analyses were adjusted for age, gender, race, education, marital status, and income.

**Table 2:** Associations between types of direct and indirect trauma and PTSD*.

	Exposure	Category	PTSD	
			OR (95% CI)	P
Model 1	Cumulative Harvey direct trauma	0	1	NA
		1–2	3.18 (1.85, 5.46)	**< .001**
Model 2	Cumulative Harvey indirect trauma	0	1	NA
		1–3	1.91 (1.05, 3.46)	**.033**
Model 3	Cumulative COVID direct trauma	0	1	NA
		1–4	2.13 (1.20, 3.77)	**.010**
Model 4	Cumulative COVID indirect trauma	0–1	1	NA
		2–3	1.69 (0.93, 3.09)	.088

*All Models adjusted for age, gender, race, education, marital status and income; Full models shown in S2 Table.

**Table 3:** Associations between cumulative direct and indirect trauma and PTSD*.

	Exposure	Category	PTSD	
			OR (95% CI)	P
Model 1	Cumulative Harvey and COVID direct trauma	0	1	NA
		1–6	2.53 (1.36, 4.70)	**.003**
Model 2	Cumulative Harvey and COVID indirect trauma	0–1	1	NA
		2–6	2.79 (1.47, 5.28)	**.002**

* All Models adjusted for age, gender, race, education, marital status and income; Full models shown in S4 Table.

All analyses were performed using SAS 9.4 program, incorporating complex survey weights, with statistical significance set at p<0.05.

The study received ethical approval from the institutional review board at Boston University Medical Campus, and all research methods adhered to the relevant guidelines and regulations. Consent was waived by the ethics committee.

## Results

As shown in Table 1, the majority of study participants identified as either male (48.1%) or female (50.5%), with a small minority (1.4%) selecting another gender category. More than half of the participants (52.1%) had attained some college education or higher, and approximately half (50.2%) were married. The sample consisted of 43.2% Hispanic individuals, 36.6% White non-Hispanic individuals, 14.2% Black non-Hispanic individuals, and 6% individuals from other non-Hispanic racial backgrounds. The average age of participants was 46.1 years. A substantial proportion of participants (61.2%) indicated a current income of less than $75,000. The interview mode was evenly split between paper (i.e., mail) (48.1%) and web (51.9%). 12.6% of participants reported experiencing current PTSD.

Descriptive analysis of the data, shown in S1 Table, reveals that a substantial portion of participants experienced a variety of exposures. For Hurricane Harvey, this included direct traumas, such as worrying about being seriously injured or killed (34.6%), and indirect trauma indirect trauma involving concerns about the serious injury or death of family members or close friends (54.8%). Furthermore, a significant number of participants were exposed to COVID-related events, including direct trauma like the fear of contracting COVID (48.5%), and indirect trauma involving concerns that family members or close friends might die from COVID (64.6%), or the experience of having seriously ill family members or friends (39.6%) or losing family members and friends due to COVID (19.4%).

We noted an increased incidence of PTSD among individuals who experienced specific Hurricane Harvey exposures, including worry about serious injury or death during Harvey (direct trauma, 22.1%), and the death of family members or close friends (indirect trauma, 32.5%). Further, we noted a higher prevalence of PTSD among those who experienced certain COVID-related traumas, including hospitalization (direct trauma, 80.4%), and the death of family members or friends due to the pandemic (indirect trauma, 17.5%).

We found a greater likelihood of PTSD when individuals were exposed to a higher number of direct or indirect traumas, in accordance with the DSM-5 criteria. Prevalence and median splits are presented in S1 Table.

Table 2 shows the results of multiple logistic regression analyses examining the relationship between direct and indirect trauma exposure for each event and PTSD prevalence. These adjusted results reveal a statistically significant relationship between direct and indirect traumas related to Hurricane Harvey and COVID-19, and the presence of PTSD. These relationships were statistically significant in all categories except for cumulative COVID indirect trauma. Associations with PTSD were demonstrated for Harvey’s direct traumas (OR = 3.18, 95% CI 1.85, 5.46), and COVID direct traumas (OR = 2.13, 95% CI 1.20, 3.77), and to a lesser extent for Harvey’s indirect trauma (OR = 1.91, 95% CI 1.05, 3.46), as presented in Table 2. The point estimates are higher for direct compared to indirect trauma, although the confidence intervals overlap. The full models are presented in S2 Table. Estimates from the unadjusted models are presented in S3 Table.

Table 3 shows the OR for PTSD for cumulative exposure to the indirect traumas of Harvey and COVID (OR = 2.79, 95% CI 1.47, 5.28) and cumulative exposure to the direct traumas of these events (OR = 2.53, 95% CI 1.36, 4.70). The full models are presented in S4 Table. Data from the unadjusted model can be found in S5 Table.

## Discussion

Our study demonstrates associations between exposure to both direct and indirect traumas resulting from large scale potentially traumatic events and the development of PTSD. Further, our data establishes associations between cumulative exposures to different large scale potentially traumatic events and the occurrence of PTSD.

In our study, approximately one out of eight participants (12.6%) fulfilled the criteria for current PTSD, three years after Hurricane Harvey and during the COVID pandemic. These data align with other studies examining PTSD among persons exposed to traumatic events using DSM criteria [4]. Additionally, our findings are consistent with other studies investigating the long-term consequences of natural disasters that occurred years earlier. For example, Raker et al. reported a 16.7% rate of post-traumatic symptoms in communities exposed to Hurricane Katrina 12 years after the event [46]. In a longitudinal study conducted by Goenjian et al., a PTSD rate of 11.6%, as per DSM-5 criteria, was identified among survivors of the Spitak earthquake in Armenia, 23 years after the incident [47]. Horesh et al. revealed a 16.5% rate of PTSD among veterans from Israel 20 years after experiencing the Lebanon war [48].

We showed an association between exposure to both direct and indirect traumas and PTSD. This finding supports the notion, consistent with other studies, that the inclusion of exposure to indirect trauma in the DSM-5 criteria was warranted. May and Wisco’s review suggests that indirect exposure to trauma among close associates, aligning with the definition used in our study, may lead to PTSD, albeit with a lower likelihood compared to direct exposure [49]. Solomon and Zerach’s analysis of indirect exposures to trauma among family members of former Israeli prisoners of war who were exposed to war trauma and subsequently developed PTSD, provides evidence of the development of secondary traumatization among spouses and offspring [50]. Common mechanisms for both direct and indirect trauma are also evident in the study by Ten Holt et al., which found associations between both direct and indirect exposure to trauma and PTSD among patients with substance use disorder [12].

Our observation that persons directly affected by traumatic events consistently exhibited a higher prevalence of PTSD compared to those indirectly affected aligns with current literature, underscoring the intensity of event exposure as a significant risk factor for PTSD. Galea et al. demonstrated the impact of indirect exposure to the September 11 terror attacks on New York residents in relation to the development of PTSD, albeit to a lesser extent compared to individuals directly affected by the attacks [13]. In contrast, Kar et al. found no significant difference in PTSD prevalence among people directly or indirectly exposed to the 2004 Asian Tsunami disaster. However, their study defined indirect exposure as being in close proximity to the sea and witnessing all the devastation and trauma around, without specifying indirect exposure through affected loved ones [14].

Findings regarding the influence of indirect exposure to trauma on the development of PTSD are not conclusive in the literature. Merdjanhoff et al. found that indirect exposure to Hurricane Katrina at the community level was notably linked to psychological distress but did not show a significant association with probable PTSD [51]. However, this study did not define indirect exposure in alignment with the DSM PTSD criteria. Instead, it included stressors in the indirect exposure category, such as economic loss and lost income. Further, some studies have identified distinct features associated with direct and indirect exposure to traumas. For example, Christou-Ergos et al. demonstrated in Australia that individuals who directly experienced significant human suffering were less inclined to accept the COVID vaccine during the pandemic, whereas those with an indirect exposure to severe suffering showed a higher willingness to receive the vaccine [52].

Our study demonstrates a cumulative effect of exposure to multiple traumas on the development of PTSD. These findings are consistent with other studies that have explored the impact of exposure to multiple traumatic events. Schock et al. observed such a cumulative effect among refugees in Germany who had been exposed to various traumatic events [23]. Lowe et al. identified a cumulative effect of exposure to two disasters in the United States, namely Hurricane Katrina and the Deepwater Horizon oil spill [53]. Harville et al. showed that exposure to multiple hurricanes among United States Gulf Coast residents increased the risk of mental health issues, including PTSD [54]. Yehuda et al. found that Holocaust survivors with PTSD had experienced significantly more traumas in life compared to survivors without PTSD [55]. These findings are also in line with other studies that have examined the combined effects of COVID-19 and natural disasters, such as the work of Agyapong et al., which revealed a higher likelihood of PTSD and other mental health disorders among people in Canada who had experienced both COVID-19 and traumas like floods or wildfires [56].

We demonstrated a cumulative effect of indirect traumas on the risk of PTSD. Such effect is supported by other studies that have investigated the cumulative mechanisms of indirect trauma exposures. Fernandez et al. discovered that patients exposed to various disaster-related experiences in the New York area in 2001, including the World Trade Center disaster and the Flight 587 crash two months later, had poorer health status compared to those exposed to only one disaster. This also involved indirect experiences related to the deaths of friends and family in these events [57]. Garfin et al. found that prior indirect exposure to three community traumas in the northern United States through the media increased the likelihood of citizens in the Boston and New York areas reacting more negatively to the Boston Marathon Bombing, which was a subsequent community trauma [58].

### Limitations

Our study has several limitations. First, the analysis for HTRS findings does not include individuals who were residing outside of Houston during the hurricane. Second, the study had a relatively low response rate of 11.4%. However, this response rate is typical for community surveys that do not offer substantial compensation to respondents [59], and despite the response rate, adjustments were made for unit non-response using a weighting approach. Third, the study is cross-sectional in nature, and therefore cannot establish causal effects. However, the odds ratio analysis provides insights into the impact of exposures to direct and indirect trauma on PTSD. Fourth, the scales utilized to assess PTSD in our study are self-report measures and not clinical diagnoses. However, we employed the PTSD Checklist for DSM-5, along with rigorous algorithm [45], to identify probable cases of current PTSD. Fifth, several other social components besides traumatic exposure may contribute to the development of PTSD, which haven’t been included in our analysis. These factors may include pre-existing mental conditions, such as acute stress disorder, anxiety sensitivity or baseline pain. However, our study focuses on traumatic events, which are the core experiences influencing the development of PTSD. And sixth, the questionnaire documents PTSD symptoms experienced during Hurricane Harvey. However, these symptoms may have originated earlier and may have been exacerbated by or reemerged in the context of the hurricane. This aligns with our interest in documenting the overall burden of PTSD following the Hurricane.

### Implications

Despite these limitations, our findings underscore the need for further investigation into the particular trauma attributes that contribute to the onset of PTSD. They highlight the necessity for a comprehensive exploration of the mechanisms connecting both direct and indirect exposures to trauma, as well as the cumulative impact of multiple traumas on the manifestation of PTSD symptoms. Additionally, our findings support the incorporation of a cumulative effect, which is currently absent from the DSM’s PTSD criteria.

Our results emphasize the importance of screening individuals affected by disasters for prior exposures to traumas, as well as screening those indirectly exposed to traumatic events, as both groups are at risk for the development of PTSD.

Further, our findings have implications for planning PTSD services, underscoring the need to develop specific programs targeting individuals exposed to indirect traumas, as well as those exposed to cumulative traumas, as vulnerable populations at risk of developing PTSD. These recommendations are particularly important in a time of compounding disasters and broad population exposures to these events [4, 60], and in light of studies that have identified other implications of trauma exposure, including psychiatric conditions like depression [61] and physical illnesses such as cardiovascular diseases [62].

The introduction of intervention programs for those exposed to cumulative traumas receives further support from studies revealing consequences for such exposures beyond PTSD. For example, Ruglass et al. found that cumulative exposure to traumatic events among American minority women increased the odds of being arrested due to criminal involvement [63]. This is particularly important given the emerging discussion in the scientific literature regarding efforts to proactively screen populations at risk for PTSD, driven by advancing research on preventive measures for the disorder [64].

## Conclusions

Our study provides compelling evidence supporting the links between exposure to both direct and indirect traumas resulting from disasters and the onset of PTSD. Additionally, it firmly echoes evidence supporting relationships between cumulative exposures to various disasters and the emergence of PTSD [27–32].

The findings concerning the influence of cumulative exposure to indirect traumas on PTSD underscore the vital role of indirect experiences in the development of PTSD. This highlights the importance of screening individuals who have been indirectly exposed to traumas involving family members or loved ones, and further emphasizes the necessity of creating intervention programs customized for these individuals.

## Supporting information

S1 Table Trauma subvariables, prevalence and median split. (DOCX)

S2 Table Associations between types of direct and indirect trauma and PTSD, unadjusted. (DOCX)

S3 Table Associations between cumulative direct and indirect trauma and PTSD, unadjusted. (DOCX)

S4 Table Associations between cumulative direct and indirect trauma and PTSD. (DOCX)

S5 Table Associations between cumulative direct and indirect trauma and PTSD, unadjusted. (DOCX)

## Abbreviations

DSM: Diagnostic and Statistical Manual of Mental Disorders
HTRS: Houston Trauma and Recovery Study
PTSD: Post Traumatic Stress Disorder

## References

[1] Hoppen TH, Priebe S, Vetter I, et al. Global burden of post-traumatic stress disorder and major depression in countries affected by war between 1989 and 2019: a systematic review and meta-analysis. BMJ Glob Health. 2021 Jul;6(7):e006303. doi: 10.1136/bmjgh-2021-006303 34321235 PMC8319986

[2] Koenen KC, Ratanatharathorn A, Ng L, McLaughlin KA, et al. Posttraumatic stress disorder in the World Mental Health Surveys. Psychol Med. 2017 Oct;47(13):2260–2274. doi: 10.1017/S0033291717000708 28385165 PMC6034513

[3] Solomon Z, Mikulincer M. Trajectories of PTSD: a 20-year longitudinal study. Am J Psychiatry. 2006 Apr;163(4):659–666. doi: 10.1176/ajp.2006.163.4.659 16585441

[4] Breslau N. The epidemiology of trauma, PTSD, and other posttrauma disorders. Trauma Violence Abuse. 2009 Jul;10(3):198–210. doi: 10.1177/1524838009334448 19406860

[5] Committee on the Assessment of Ongoing Effects in the Treatment of Posttraumatic Stress Disorder; Institute of Medicine. Treatment for Posttraumatic Stress Disorder in Military and Veteran Populations: Initial Assessment. Washington (DC): National Academies Press (US); 2012 Jul 13 [cited 2023 Oct 9]. 2, History, Diagnostic Criteria, and Epidemiology. https://www.ncbi.nlm.nih.gov/books/NBK201095/24830058

[6] Turnbull GJ. A review of post-traumatic stress disorder. Part I: Historical development and classification. Injury. 1998 Mar;29(2):87–91. doi: 10.1016/s0020-1383(97)00131-9 10721399

[7] Solomon Z, Horesh D, Ginzburg K. Trajectories of PTSD and secondary traumatization: A longitudinal study. J Psychiatr Res. 2021 Jun;138:354–359. doi: 10.1016/j.jpsychires.2021.03.027 33930614

[8] Bromet EJ, Atwoli L, Kawakami N, et al. Post-traumatic stress disorder associated with natural and human-made disasters in the World Mental Health Surveys. Psychol Med. 2017 Jan;47(2):227–241. doi: 10.1017/S0033291716002026 27573281 PMC5432967

[9] Galea S, Nandi A, Vlahov D. The epidemiology of post-traumatic stress disorder after disasters. Epidemiol Rev. 2005;27:78–91. doi: 10.1093/epirev/mxi003 15958429

[10] Figley CR. Compassion fatigue as secondary traumatic stress disorder: An overview. In: Figley CR, editor. Compassion fatigue: Coping with secondary traumatic stress disorder in those who treat the traumatized. New York, NY: Brunner/Mazel, 1995. pp. 1–20.

[11] Greinacher A, Nikendei A, Kottke R, et al. Secondary Traumatization, Psychological Stress, and Resilience in Psychosocial Emergency Care Personnel. Int J Environ Res Public Health. 2019 Sep 3;16(17):3213. doi: 10.3390/ijerph16173213 31484307 PMC6747539

[12] Ten Holt J, van Emmerik AAP, Blanken P. et al. Direct and indirect exposure to trauma, posttraumatic stress disorder symptoms, and poor subjective sleep quality in patients with substance use disorder. Psychiatry Clin Psychopharmacol. 2022;32(3):188–195. doi: 10.5152/pcp.2022.22368 38766672 PMC11099636

[13] Galea S, Vlahov D, Resnick H, et al. Trends of probable post-traumatic stress disorder in New York City after the September 11 terrorist attacks. Am J Epidemiol. 2003 Sep 15;158(6):514–524. doi: 10.1093/aje/kwg187 12965877

[14] Kar N, Krishnaraaj R, Rameshraj K. Long-term mental health outcomes following the 2004 Asian tsunami disaster: A comparative study on direct and indirect exposure. Disaster Health. 2013 Apr 17;2(1):35–45. doi: 10.4161/dish.24705 28228999 PMC5314937

[15] American Psychiatric Association. Diagnostic and Statistical Manual of Mental Disorders. 5th ed. 2013.

[16] Friedman MJ. Finalizing PTSD in DSM-5: getting here from there and where to go next. J Trauma Stress. 2013 Oct;26(5):548–556. doi: 10.1002/jts.21840 24151001

[17] Abdalla SM, Cohen GH, Tamrakar S, et al. Media Exposure and the Risk of Post-Traumatic Stress Disorder Following a Mass traumatic Event: An In-silico Experiment. Front Psychiatry. 2021 Nov 25;12:674263. doi: 10.3389/fpsyt.2021.674263 34899406 PMC8656276

[18] Hall BJ, Xiong YX, Yip PSY, et al. The association between disaster exposure and media use on post-traumatic stress disorder following Typhoon Hato in Macao, China. Eur J Psychotraumatol. 2019 Jan 14;10(1):1558709. doi: 10.1080/20008198.2018.1558709 30693078 PMC6338284

[19] Palgi Yuval & Shrira Amit & Hoffman Yaakov. (2017). Negative and positive perceptions of media sources and PTSD symptoms among older adults exposed to missile attacks. Personality and Individual Differences. 119. 185–188. doi: 10.1016/j.paid.2017.07.025

[20] Spitzer RL, First MB, Wakefield JC. Saving PTSD from itself in DSM-V. J Anxiety Disord. 2007;21(2):233–241. doi: 10.1016/j.janxdis.2006.09.006 17141468

[21] McNally RJ. Can we fix PTSD in DSM-V? Depress Anxiety. 2009;26(7):597–600. doi: 10.1002/da.20586 19569228

[22] Wilker S, Pfeiffer A, Kolassa S, Koslowski D, Elbert T, Kolassa IT. How to quantify exposure to traumatic stress? Reliability and predictive validity of measures for cumulative trauma exposure in a post-conflict population. Eur J Psychotraumatol. 2015 Nov 19;6:28306. doi: 10.3402/ejpt.v6.28306 26589255 PMC4654773

[23] Schock K, Böttche M, Rosner R, Wenk-Ansohn M, Knaevelsrud C. Impact of new traumatic or stressful life events on pre-existing PTSD in traumatized refugees: results of a longitudinal study. Eur J Psychotraumatol. 2016 Nov 9;7:32106. doi: 10.3402/ejpt.v7.32106 27834172 PMC5105333

[24] Breslau N, Chilcoat HD, Kessler RC, et al. Previous exposure to trauma and PTSD effects of subsequent trauma: results from the Detroit Area Survey of Trauma. Am J Psychiatry. 1999 Jun;156(6):902–907. doi: 10.1176/ajp.156.6.902 10360130

[25] Kaysen D, Resick PA, Wise D. Living in danger: the impact of chronic traumatization and the traumatic context on posttraumatic stress disorder. Trauma Violence Abuse. 2003 Jul;4(3):247–264. doi: 10.1177/1524838003004003004 14697125

[26] Kube T, Elssner AC, Herzog P. The relationship between multiple traumatic events and the severity of posttraumatic stress disorder symptoms—evidence for a cognitive link. Eur J Psychotraumatol. 2023;14(1):2165025. doi: 10.1080/20008066.2023.2165025 37052097 PMC9879173

[27] Post RM. Transduction of psychosocial stress into the neurobiology of recurrent affective disorder. Am J Psychiatry. 1992 Aug;149(8):999–1010. doi: 10.1176/ajp.149.8.999 1353322

[28] da Silva HC, Vilete L, Coutinho ESF, et al. The role of childhood cumulative trauma in the risk of lifetime PTSD: An epidemiological study. Psychiatry Res. 2024;336:115887. doi: 10.1016/j.psychres.2024.115887 38642421

[29] Garon-Bissonnette J, Bolduc MG, Lemieux R, et al. Cumulative childhood trauma and complex psychiatric symptoms in pregnant women and expecting men. BMC Pregnancy Childbirth. 2022;22(1):10. doi: 10.1186/s12884-021-04327-x 34983417 PMC8725451

[30] Gonzalez A, Monzon N, Solis D, et al. Trauma exposure in elementary school children: Description of screening procedures, level of exposure, and posttraumatic stress symptoms. School Ment Health. 2016 Mar;8(1):77–88. doi: 10.1007/s12310-015-9167-7 27721907 PMC5049495

[31] Park J, Jun JY, Lee YJ, et al. The association between alexithymia and posttraumatic stress symptoms following multiple exposures to traumatic events in North Korean refugees. J Psychosom Res. 2015 Jan;78(1):77–81. doi: 10.1016/j.jpsychores.2014.09.007 25248674

[32] Gerber M, Frankfurt SB, Contractor AA, et al. Influence of Multiple Traumatic Event Types on Mental Health Outcomes: Does Count Matter?. J Psychopathol Behav Assess. 2018;40:645–654. doi: 10.1007/s10862-018-9682-6

[33] Li X, Fu D, Nielsen-Gammon J, et al. Impacts of climate change on future hurricane induced rainfall and flooding in a coastal watershed: A case study on Hurricane Harvey. J Hydrol. 2023;616:N.PAG. doi: 10.1016/j.hydrol.2022.128774

[34] Blake ES, Zelinsky DA. National Hurricane Center Tropical Cyclone Report: Hurricane Harvey. 2017. [cited 2023 Oct 9]. chrome-extension://efaidnbmnnnibpcajpcglclefindmkaj/https://www.nhc.noaa.gov/data/tcr/AL092017_Harvey.pdf

[35] Seddighi H. COVID-19 as a Natural Disaster: Focusing on Exposure and Vulnerability for Response. Disaster Med Public Health Prep. 2020 Aug;14(4):e42–e43. doi: 10.1017/dmp.2020.279 32713408 PMC7492580

[36] World Health Organization. Coronavirus Disease (COVID-19) Pandemic. [cited 2023 Oct 9]. https://www.who.int/emergencies/diseases/novel-coronavirus-2019?adgroupsurvey={adgroupsurvey}&gclid=CjwKCAjwr_CnBhA0EiwAci5sin297QiN4TjgRf6Sktlz0AmOlue_LfmPOYVVzXgXGT74lQFBltewfRoCPZ4QAvD_BwE

[37] Abdalla SM, Ettman CK, Cohen GH, et al. Mental health consequences of COVID-19: a nationally representative cross-sectional study of pandemic-related stressors and anxiety disorders in the USA. BMJ Open. 2021 Aug 9;11(8):e044125. doi: 10.1136/bmjopen-2020-044125 34373289 PMC8354762

[38] Serra R, Borrazzo C, Vassalini P, et al. Post-Traumatic Stress Disorder Trajectories the Year after COVID-19 Hospitalization. Int J Environ Res Public Health. 2022 Jul 11;19(14):8452. doi: 10.3390/ijerph19148452 35886306 PMC9316829

[39] Carmassi C, Foghi C, Dell’Oste V, et al. PTSD symptoms in healthcare workers facing the three coronavirus outbreaks: What can we expect after the COVID-19 pandemic. Psychiatry Res. 2020 Oct;292:113312. doi: 10.1016/j.psychres.2020.113312 32717711 PMC7370915

[40] City of Houston. Super Neighborhoods. [cited 2023 Oct 9]. http://www.houstontx.gov/superneighborhoods/.

[41] United States Census Bureau. American Community Survey Information Guide. [cited 2023 Oct 9]. https://www.census.gov/programs-surveys/acs/library/information-guide.html

[42] Rubin DB. Multiple imputation for nonresponse in surveys, vol. 81. Wiley. 2004.

[43] Mol SS, Arntz A, Metsemakers JF, et al. Symptoms of post-traumatic stress disorder after non-traumatic events: evidence from an open population study. Br J Psychiatry. 2005 Jun;186:494–9. doi: 10.1192/bjp.186.6.494 15928360

[44] Bovin MJ, Marx PB, Weathers TW, et al. Psychometric properties of the PTSD checklist for diagnostic and statistical manual of mental disorders–fifth edition (PCL-5) in veterans. Psychol Assess. 2016;28(11):1379–1391. doi: 10.1037/pas0000254 26653052

[45] Hoge CW, Riviere LA, Wilk JE, et al. The prevalence of post-traumatic stress disorder (PTSD) in US combat soldiers: a head-to-head comparison of DSM-5 versus DSM-IV-TR symptom criteria with the PTSD checklist. Lancet Psychiatry. 2014;1(4):269–277. doi: 10.1016/S2215-0366(14)70235-4 26360860

[46] Raker EJ, Lowe SR, Arcaya MC, et al. Twelve years later: The long-term mental health consequences of Hurricane Katrina. Soc Sci Med. 2019 Dec;242:112610. doi: 10.1016/j.socscimed.2019.112610 31677480 PMC8450020

[47] Goenjian AK, Khachadourian V, Armenian H, et al. Posttraumatic Stress Disorder 23 Years After the 1988 Spitak Earthquake in Armenia. J Trauma Stress. 2018 Feb;31(1):47–56. doi: 10.1002/jts.22260 29513918

[48] Horesh D, Solomon Z, Keinan G, et al. The clinical picture of late-onset PTSD: a 20-year longitudinal study of Israeli war veterans. Psychiatry Res. 2013 Aug 15;208(3):265–273. doi: 10.1016/j.psychres.2012.12.004 23294854

[49] May CL, Wisco BE. Defining trauma: How level of exposure and proximity affect risk for posttraumatic stress disorder. Psychol Trauma. 2016;8(2),233–240. doi: 10.1037/tra0000077 26390110

[50] Solomon Z, Zerach G. The Intergenerational transmission of trauma: When children bear their father’s traumatic past. Neuropsychiatr Enfance Adolesc. 2020;68(2), 65–75. doi: 10.1016/j.neurenf.2020.01.004

[51] Merdjanoff A, Abramson D, Piltch-Loeb R, et al. Examining the Dose–Response Relationship: Applying the Disaster Exposure Matrix to Understand the Mental Health Impacts of Hurricane Sandy. Clin Soc Work J. 2021;50. doi: 10.1007/s10615-021-00814-y

[52] Christou-Ergos M, Wiley KE, Leask J. Association between traumatic life events and vaccine hesitancy: A cross-sectional Australian study. Public Health. 2023 Mar;216:1–6. doi: 10.1016/j.puhe.2022.12.008 36669258

[53] Lowe SR, McGrath JA, Young MN, et al. Cumulative Disaster Exposure and Mental and Physical Health Symptoms Among a Large Sample of Gulf Coast Residents. J Trauma Stress. 2019 Apr;32(2):196–205. doi: 10.1002/jts.22392 30913348 PMC6476642

[54] Harville EW, Xiong X, Smith BW, et al. Combined effects of Hurricane Katrina and Hurricane Gustav on the mental health of mothers of small children. J Psychiatr Ment Health Nurs. 2011 May;18(4):288–296. doi: 10.1111/j.1365-2850.2010.01658.x 21418428 PMC3472438

[55] Yehuda R, Kahana B, Schmeidler J, et al. Impact of cumulative lifetime trauma and recent stress on current posttraumatic stress disorder symptoms in holocaust survivors. Am J Psychiatry. 1995 Dec;152(12):1815–1818. doi: 10.1176/ajp.152.12.1815 8526254

[56] Agyapong B, Shalaby R, Eboreime E, et al. Cumulative trauma from multiple natural disasters increases mental health burden on residents of Fort McMurray. Eur J Psychotraumatol. 2022 May 17;13(1):2059999. doi: 10.1080/20008198.2022.2059999 35599978 PMC9116266

[57] Fernandez WG, Galea S, Miller J, et al. Health status among emergency department patients approximately one year after consecutive disasters in New York City. Acad Emerg Med. 2005 Oct;12(10):958–964. doi: 10.1197/j.aem.2005.06.005 16204139

[58] Garfin DR, Holman EA, Silver RC. Cumulative exposure to prior collective trauma and acute stress responses to the Boston marathon bombings. Psychol Sci. 2015 Jun;26(6):675–83. doi: 10.1177/0956797614561043 25896419

[59] Sinclair M., O’Toole J., Malawaraarachchi M. et al. Comparison of response rates and cost-effectiveness for a community-based survey: postal, internet and telephone modes with generic or personalised recruitment approaches. BMC Med Res Methodol. 2012;12:132. doi: 10.1186/1471-2288-12-132 22938205 PMC3502082

[60] Benjet C, Bromet E, Karam EG, et al. The epidemiology of traumatic event exposure worldwide: results from the World Mental Health Survey Consortium. Psychol Med. 2016 Jan;46(2):327–343. doi: 10.1017/S0033291715001981 26511595 PMC4869975

[61] Cohen GH, Wang R, Sampson L, et al. Depression and PTSD among Houston Residents who Experienced Hurricane Harvey and COVID-19: Implications for Urban Areas Affected by Multiple Disasters. J Urban Health. 2023;100(4):860–869. doi: 10.1007/s11524-023-00767-2 37550501 PMC10447846

[62] Sumner J.A., Cleveland S., Chen T. et al. Psychological and biological mechanisms linking trauma with cardiovascular disease risk. Transl Psychiatry. 2023;13:25. doi: 10.1038/s41398-023-02330-8 36707505 PMC9883529

[63] Ruglass LM, Espinosa A, Sykes KM, et al. Direct and Indirect Effects of Cumulative Trauma, PTSD, and Substance Use Disorder on Probability of Arrest Among Lower Income African American and Latina Women. Race Justice. 2018;8(2), 126–153. doi: 10.1177/2153368716656917

[64] Magwood O, Bellai-Dussault K, Fox G, et al. Diagnostic test accuracy of screening tools for post-traumatic stress disorder among refugees and asylum seekers: A systematic review and meta-analysis. J Migr Health. 2022 Dec 10;7:100144. doi: 10.1016/j.jmh.2022.100144 36568829 PMC9772565

