# Supplementary material for: The cumulative contribution of direct and indirect traumas to the production of PTSD
Source: PLoS One. 2024 Aug 14;19(8):e0307593. doi: 10.1371/journal.pone.0307593 (PMC11324107; doi:10.1371/journal.pone.0307593)
Supplement: S1 Table — (DOCX) [file pone.0307593.s001.docx]

S1 Table: Trauma subvariables, prevalence and median split

| Exposure | Category | n (%) | DSM-5 criteria  n (%) |
| --- | --- | --- | --- |
| Seriously injured | Yes | 10 (1.0) | 2 (16.9) |
|  | No | 1157 (99.0) | 135 (12.6) |
| Worried of seriously injured or killed | Yes | 388 (34.6) | 78 (22.1) |
|  | No | 779 (65.4) | 59 (7.6) |
| Cumulative Harvey Direct Trauma | 0 | 775 (65.1) | 59 (7.6) |
|  | 1-2 | 392 (34.9) | 78 (22.0) |
| Family member or close friend seriously injured | Yes | 44 (4.6) | 6 (7.4) |
|  | No | 1123 (95.4) | 131 (12.9) |
| Family member or close friend killed | Yes | 21 (2.2) | 7 (32.5) |
|  | No | 1146 (97.8) | 130 (12.2) |
| Worried of family members or close friend seriously injured or killed | Yes | 595 (54.8) | 98 (17.0) |
|  | No | 572 (45.2) | 39 (7.3) |
| Cumulative Harvey Indirect Trauma | 0 | 557 (44.0) | 37 (7.5) |
|  | 1-3 | 610 (56.0) | 100 (16.7) |
| Became seriously ill | Yes | 65 (9.0) | 18 (33.7) |
|  | No | 912 (91.0) | 90 (10.0) |
| Hospitalized | Yes | 10 (1.6) | 5 (80.4) |
|  | No | 967 (98.4) | 103 (11.1) |
| Worried of getting COVID | Yes | 456 (48.5) | 56 (13.2) |
|  | No | 477 (51.5) | 43 (8.9) |
| Thought of dying from COVID | Yes | 32 (5.1) | 12 (38.0) |
|  | No | 945 (94.5) | 96 (10.8) |
| Cumulative COVID Direct Trauma | 0 | 623 (54.7) | 60 (9.2) |
|  | 1-4 | 522 (45.3) | 75 (16.6) |
| Had seriously ill family members/ close friends | Yes | 440 (39.6) | 66 (16.7) |
|  | No | 727 (60.4) | 71 (9.9) |
| Had family members/ close friends die from COVID | Yes | 224 (19.4) | 37 (17.5) |
|  | No | 943 (80.6) | 100 (11.5) |
| Worried family members/ close friends might die from COVID | Yes | 688 (64.6) | 97 (14.3) |
|  | No | 479 (35.4) | 40 (9.6) |
| Cumulative COVID Indirect Trauma | 0-1 | 798 (65.1) | 79 (10.4) |
|  | 2-3 | 369 (34.9) | 58 (16.9) |
| Cumulative Harvey and COVID Direct Trauma | 0 | 470 (40.8) | 33 (6.8) |
|  | 1-6 | 697 (59.2) | 104 (16.6) |
| Cumulative Harvey and COVID Indirect Trauma | 0-1 | 552 (41.6) | 39 (6.0) |
|  | 2-6 | 615 (58.4) | 98 (17.4) |
