# Supplementary material for: The cumulative contribution of direct and indirect traumas to the production of PTSD
Source: PLoS One. 2024 Aug 14;19(8):e0307593. doi: 10.1371/journal.pone.0307593 (PMC11324107; doi:10.1371/journal.pone.0307593)
Supplement: S2 Table — (DOCX) [file pone.0307593.s002.docx]

S2 Table: Associations between types of direct and indirect trauma and PTSD*

| Exposure | Category | Model 1 | | Model 2 | | Model 3 | | Model 4 | |
| --- | --- | --- | --- | --- | --- | --- | --- | --- | --- |
|  |  | OR  (95% CI) | P | OR  (95% CI) | P | OR  (95% CI) | P | OR  (95% CI) | P |
| Age | 18-99 | \| 0.99  (0.97, 1.01) \| \| --- \| | 0.351 | 0.99  (0.97, 1.01) | 0.494 | 0.99  (0.97, 1.01) | 0.333 | 0.99  (0.97, 1.01) | 0.466 |
| Gender | Male | 1 | NA | 1 | NA | 1 | NA | 1 | NA |
|  | Female | 0.92  (0.51, 1.67) | 0.785 | 0.98  (0.54, 1.78) | 0.954 | 1.01  (0.56, 1.83) | 0.971 | 0.98  (0.54, 1.78) | 0.955 |
|  | Other | 12.84  (2.42, 68.26) | 0.003 | 9.12  (1.83, 45.58) | 0.007 | 10.06  (2.36, 42.92) | 0.002 | 10.70  (2.24, 51.04) | 0.003 |
| Race | White, non-Hispanic | 1 | NA | 1 | NA | 1 | NA | 1 | NA |
|  | Black, non-Hispanic | 3.14  (1.30, 7.55) | 0.011 | 3.40  (1.42, 8.15) | 0.006 | 4.20  (1.74, 10.15) | 0.001 | 3.60  (1.52, 8.53) | 0.004 |
|  | Other or 2+ Races, non-Hispanic | 1.58  (0.40, 6.22) | 0.510 | 1.64  (0.41, 6.49) | 0.483 | 1.80  (0.45, 7.18) | 0.401 | 1.58  (0.40, 6.35) | 0.514 |
|  | Hispanic | 1.31  (0.55, 3.13) | 0.546 | 1.52  (0.64, 3.63) | 0.341 | 1.62  (0.69, 3.82) | 0.270 | 1.52  (0.61, 3.79) | 0.365 |
| Education | High school or less | 0.73  (0.39, 1.37) | 0.328 | 0.79  (0.42, 1.49) | 0.474 | 0.84  (0.46, 1.56) | 0.585 | 0.89  (0.47, 1.66) | 0.707 |
|  | Some college or  higher | 1 | NA | 1 | NA | 1 | NA | 1 | NA |
| Marital Status | Divorced, separated, or widowed | 2.28  (1.07, 4.85) | 0.033 | 2.22  (1.06, 4.67) | 0.036 | 2.37  (1.14, 4.92) | 0.021 | 2.40  (1.15, 5.03) | 0.020 |
|  | Never married | 1.31  (0.61, 2.82) | 0.488 | 1.38  (0.63, 2.98) | 0.420 | 1.34  (0.61, 2.94) | 0.466 | 1.37  (0.63, 3.00) | 0.426 |
|  | Married | 1 | NA | 1 | NA | 1 | NA | 1 | NA |
| Income | ≤$24,999 | 3.55  (0.92, 13.70) | 0.066 | 3.15  (0.87, 11.46) | 0.081 | 3.21  (0.92, 11.22) | 0.067 | 3.25  (0.87, 12.15) | 0.079 |
|  | $25,000-$49,999 | 2.32  (0.61, 8.86) | 0.218 | 2.11  (0.59, 7.56) | 0.253 | 2.00  (0.57, 7.02) | 0.277 | 2.25  (0.61, 8.26) | 0.223 |
|  | $50,000-$74,999 | 1.33  (0.28, 6.36) | 0.723 | 1.21  (0.26, 5.65) | 0.807 | 1.18  (0.27, 5.26) | 0.827 | 1.38  (0.30, 6.40) | 0.684 |
|  | $75,000-$99,999 | 3.61  (0.81, 16.15) | 0.093 | 3.35  (0.80, 14.06) | 0.098 | 3.06  (0.74, 12.66) | 0.122 | 3.66  (0.85, 15.75) | 0.082 |
|  | $100,000-$149,999 | 2.02  (0.39, 10.50) | 0.404 | 1.76  (0.36, 8.56) | 0.486 | 1.81  (0.38, 8.71) | 0.458 | 1.82  (0.36, 9.18) | 0.470 |
|  | $150,000-$199,999 | 3.30  (0.72, 15.09) | 0.124 | 3.02  (0.71, 12.87) | 0.135 | 2.91  (0.70, 12.14) | 0.143 | 3.07  (0.70, 13.53) | 0.137 |
|  | ≥$200,000 | 1 | NA | 1 | NA | 1 | NA | 1 | NA |
| Cumulative Harvey direct trauma | 0 | 1 | NA | - | - | - | - | - | - |
|  | 1-2 | 3.18  (1.85, 5.46) | <0.001 | - | - | - | - | - | - |
| Cumulative Harvey indirect trauma | 0 | - | - | 1 | NA | - | - | - | - |
|  | 1-3 | - | - | 1.91  (1.05, 3.46) | 0.033 | - | - | - | - |
| Cumulative COVID direct trauma | 0 | - | - | - | - | 1 | NA | - | - |
|  | 1-4 | - | - | - | - | 2.13  (1.20, 3.77) | 0.010 | - | - |
| Cumulative COVID indirect trauma | 0-1 | - | - | - | - | - | - | 1 | NA |
|  | 2-3 | - | - | - | - | - | - | 1.69  (0.93, 3.09) | 0.088 |

*Models adjusted for age, gender, race, education, marital status and income
