# Supplementary material for: The cumulative contribution of direct and indirect traumas to the production of PTSD
Source: PLoS One. 2024 Aug 14;19(8):e0307593. doi: 10.1371/journal.pone.0307593 (PMC11324107; doi:10.1371/journal.pone.0307593)
Supplement: S3 Table — (DOCX) [file pone.0307593.s003.docx]

S3 Table: Associations between types of direct and indirect trauma and PTSD, unadjusted.

| Exposure | Category | DSM-5 criteria | |
| --- | --- | --- | --- |
|  |  | OR  (95% CI) | P |
| Cumulative Harvey direct trauma | 0 | 1 | NA |
|  | 1-2 | 3.40 (2.00,5.77) | <0.001 |
| Cumulative Harvey indirect trauma | 0 | 1 | NA |
|  | 1-3 | 2.48 (1.42, 4.34) | 0.001 |
| Cumulative COVID direct trauma | 0 | 1 | NA |
|  | 1-4 | 1.95 (1.14, 3.36) | 0.015 |
| Cumulative COVID indirect trauma | 0-1 | 1 | NA |
|  | 2-3 | 1.76 (1.02, 3.04) | 0.042 |
