# Supplementary material for: The cumulative contribution of direct and indirect traumas to the production of PTSD
Source: PLoS One. 2024 Aug 14;19(8):e0307593. doi: 10.1371/journal.pone.0307593 (PMC11324107; doi:10.1371/journal.pone.0307593)
Supplement: S4 Table — (DOCX) [file pone.0307593.s004.docx]

S4 Table: Associations between cumulative direct and indirect trauma and PTSD*

| Exposure | Category | Model 1 | | Model 2 | |
| --- | --- | --- | --- | --- | --- |
|  |  | OR  (95% CI) | P | OR  (95% CI) | P |
| Age | 18-99 | 0.99  (0.97, 1.01) | 0.371 | 0.99  (0.97, 1.01) | 0.504 |
| Gender | Male | 1 | NA | 1 | NA |
|  | Female | 0.96  (0.53, 1.74) | 0.881 | 1.01  (0.56, 1.82) | 0.985 |
|  | Other | 9.34  (2.17, 40.16) | 0.003 | 10.00  (1.94, 51.66) | 0.006 |
| Race | White, non-Hispanic | 1 | NA | 1 | NA |
|  | Black, non-Hispanic | 3.72  (1.56, 8.90) | 0.003 | 3.20  (1.35, 7.57) | 0.008 |
|  | Other or 2+ Races, non-Hispanic | 1.75  (0.43, 7.12) | 0.436 | 1.70  (0.44, 6.61) | 0.445 |
|  | Hispanic | 1.54  (0.64, 3.73) | 0.334 | 1.36  (0.56, 3.33) | 0.496 |
| Education | High school or less | 0.86  (0.47, 1.60) | 0.637 | 0.80  (0.43, 1.51) | 0.498 |
|  | Some college or  higher | 1 | NA | 1 | NA |
| Marital Status | Divorced, separated, or widowed | 2.32  (1.12, 4.82) | 0.023 | 2.27  (1.10, 4.70) | 0.027 |
|  | Never married | 1.31  (0.61, 2.82) | 0.484 | 1.34  (0.60, 2.97) | 0.471 |
|  | Married | 1 | NA | 1 | NA |
| Income | ≤$24,999 | 3.27  (0.90, 11.89) | 0.072 | 3.03  (0.82, 11.22) | 0.097 |
|  | $25,000-$49,999 | 2.14  (0.59, 7.78) | 0.250 | 2.01  (0.55, 7.32) | 0.289 |
|  | $50,000-$74,999 | 1.19  (0.26, 5.43) | 0.819 | 1.19  (0.26, 5.45) | 0.820 |
|  | $75,000-$99,999 | 3.40  (0.82, 14.20) | 0.093 | 3.48  (0.80, 15.12) | 0.097 |
|  | $100,000-$149,999 | 2.02  (0.41, 10.10) | 0.390 | 1.61  (0.33, 7.88) | 0.555 |
|  | $150,000-$199,999 | 3.03  (0.70, 13.18) | 0.139 | 2.91  (0.67, 12.66) | 0.154 |
|  | ≥$200,000 | 1 | NA | 1 | NA |
| Cumulative Harvey and COVID direct trauma | 0 | 1 | NA | - | - |
|  | 1-6 | 2.53 (1.36, 4.70) | 0.003 | - | - |
| Cumulative Harvey and COVID indirect trauma | 0-1 | - | - | 1 | NA |
|  | 2-6 | - | - | 2.79 (1.47, 5.28) | 0.002 |

* Models adjusted for age, gender, race, education, marital status and income
