# Supplementary material for: The cumulative contribution of direct and indirect traumas to the production of PTSD
Source: PLoS One. 2024 Aug 14;19(8):e0307593. doi: 10.1371/journal.pone.0307593 (PMC11324107; doi:10.1371/journal.pone.0307593)
Supplement: S5 Table — (DOCX) [file pone.0307593.s005.docx]

S5 Table: Associations between cumulative direct and indirect trauma and PTSD, unadjusted.

| Exposure | Category | DSM-5 criteria | |
| --- | --- | --- | --- |
|  |  | OR  (95% CI) | P |
| Cumulative Harvey and COVID direct trauma | 0 | 1 | NA |
|  | 1-6 | 2.71 (1.48, 4.99) | 0.001 |
| Cumulative Harvey and COVID indirect trauma | 0-1 | 1 | NA |
|  | 2-6 | 3.31 (1.80, 6.09) | <0.001 |
